# *Fusarium* Photoreceptors

**DOI:** 10.3390/jof9030319

**Published:** 2023-03-04

**Authors:** Javier Pardo-Medina, M. Carmen Limón, Javier Avalos

**Affiliations:** 1 Departamento de Genética, Facultad de Biología, Universidad de Sevilla, 41012 Sevilla, Spain; 2 Centro de Biotecnología y Genómica de Plantas, Instituto Nacional de Investigación y Tecnología Agraria y Alimentaria (INIA), Universidad Politécnica de Madrid, 28223 Madrid, Spain

**Keywords:** light detection, flavoprotein, White Collar, cryptochrome, rhodopsin, phytochrome, RNA-seq analyses

## Abstract

Light is an important modulating signal in fungi. *Fusarium* species stand out as research models for their phytopathogenic activity and their complex secondary metabolism. This includes the synthesis of carotenoids, whose induction by light is their best known photoregulated process. In these fungi, light also affects other metabolic pathways and developmental stages, such as the formation of conidia. Photoreceptor proteins are essential elements in signal transduction from light. *Fusarium* genomes contain genes for at least ten photoreceptors: four flavoproteins, one photolyase, two cryptochromes, two rhodopsins, and one phytochrome. Mutations in five of these genes provide information about their functions in light regulation, in which the flavoprotein WcoA, belonging to the White Collar (WC) family, plays a predominant role. Global transcriptomic techniques have opened new perspectives for the study of photoreceptor functions and have recently been used in *Fusarium fujikuroi* on a WC protein and a cryptochrome from the DASH family. The data showed that the WC protein participates in the transcriptional control of most of the photoregulated genes, as well as of many genes not regulated by light, while the DASH cryptochrome potentially plays a supporting role in the photoinduction of many genes.

## 1. Introduction

Light controls important physiological and morphological responses in fungi, including circadian rhythms, morphogenesis, tropism, and the synthesis of pigments, among others [1]. The integration of external signals helps organisms to improve their competitiveness in their environment and constitutes an important driving force of evolution and adaptation [2]. Most organisms, including fungi, have evolved systems to detect light and adapt their activity accordingly. After decades of studying light responses in fungi, the application of new “omics” techniques, supported by the availability of many fungal genome sequences, has provided novel insights into light sensing and signaling pathways in these organisms [3]. From a transcriptomic point of view, light regulates the expression of hundreds of genes in different fungi [4,5,6,7].

Responses to light frequently include the modulation of characteristic fungal processes, such as sporulation and the production of secondary metabolites and hydrolytic enzymes. In addition to their ecological importance, many fungi are interesting due to their outstanding metabolic diversity [8]. They are the source of valuable compounds for the chemical or food industry [9,10,11], but they also produce many toxic metabolites, known as mycotoxins [12]. Furthermore, their adaptability and resilience make them efficient pathogens that affect animal and plant biodiversity, causing epidemics in staple crops, and even affecting human health [13]. The fungi belonging to the genus *Fusarium* stand out as models in important fields of research, such as the mechanisms that control their pathogenicity, or the production of potentially beneficial or harmful compounds. Light affects the secondary metabolism and pathogenic activity of fungi, and the regulatory mechanisms involved have received much attention [14]. Therefore, light is also expected to play an important role in many aspects of the *Fusarium* life cycle.

## 2. *Fusarium* Photobiology

The genus *Fusarium* comprises a large and heterogeneous group of ascomycetes widely distributed in nature. Many of them have received attention as phytopathogens, with great impacts on crops and as mycotoxin producers, but others are non-pathogenic, endophytic, saprophytic, or parasitic species of other organisms [15,16]. The global distribution of the genus is attributed both to its metabolic diversity, which broadens its accessibility to very diverse potential substrates, and to its efficient dispersal mechanism, which is based on the production of different types of conidia [16]. The complex taxonomy of *Fusarium* species has been clarified by DNA-based phylogenetic analyses, which revealed a monophyletic lineage consisting of 20 species complexes including almost 300 phylogenetically distinct species [17]. At present, different species of the *Fusarium* genus are widely used in research, e.g., *Fusarium graminearum*, *Fusarium oxysporum*, and *Fusarium fujikuroi*, which are normally associated with pathogenesis or secondary metabolism. Some features of the biology of these species, especially those related to development and metabolite production, are influenced by light. Over 80 years, considerable progress has been made in describing *Fusarium* photoresponses, the first reports dating back to 1962 [18].

### 2.1. Effects of Light on Development

The developmental processes related to asexual reproduction in *Fusarium* are influenced by different factors and environmental cues, including light [19]. Fungi of this species spread asexually through the formation of three types of spores, macroconidia, microconidia, and chlamydospores [16]. The different kinds of spores share common characteristics in different *Fusarium* species, but there is considerable morphological diversity [20,21]. Macroconidia are long, typically sickle-shaped, with transverse septa, usually containing several cells [19]. Microconidia are usually unicellular, although they are not produced by all *Fusarium* species, and a few species can arrange microconidia in chains [16]. Chlamydospores are thick-walled cells, usually formed inside the hyphae, capable of surviving in adverse conditions and for long periods of time [22]. Conidia of either type are usually produced in abundance to promote rapid dispersal and the colonization of new habitats, including other pathogenic hosts. Due to their multinucleated nature, it can be expected that the macroconidia are more resistant than the microconidia to adverse conditions, and it has been reported that they can develop chlamydospores [23].

Visible and near-UV lights have been reported to enhance conidia production in different *Fusarium* species [24]. Thus, in *Fusarium verticillioides*, short-wavelength blue light is particularly effective in stimulating conidia production [25]. Conidiation levels and the presence of macroconidia are very variable among different *F. fujikuroi* strains [26]. Macroconidia are rarely observed in IMI58289, a widely used wild-type strain, but they are frequently found in FKMC1995, which has been used in different works described in this review. Regulatory differences are also observed between both strains. For example, light induces conidiation in IMI58289 [24,26] although it has a negative effect in FKMC1995 [27]. The differential fitness of conidia produced at different wavelengths towards light has also been described [27].

In *F. graminearum*, conidiation under near-UV light requires the *abaA* gene product [28] conserved in other fungi. The induction of conidiation by light in *F. fujikuroi* IMI58289 does not occur in mutants of the GATA factor Csm1 [29]. The latter, and other proteins, such as histone methyltransferase Set1 and demethylase Kdm5, control the expression of the key conidiation regulator gene *abaA* [30]. In this research, conidiation was tested under illumination regimes, but the effect of light was not investigated. The elimination of *Fvve1* in *F. verticillioides* alters the aerial development of the hyphae and reduces their hydrophobicity, and in submerged cultures it activates conidiation. Interestingly, this mutation increases the ratio of macroconidia to microconidia [31]. The relationship of this protein with light regulation (see Section 2.2) provides a possible explanation for the influence of light on conidiation. The possible involvement of photoreceptors in conidiation, investigated through the effect of their gene mutations, is mentioned in Section 4, Section 5, Section 6 and Section 7.

Regulation by light also involves sexual reproduction. In sexually competent species, the formation of perithecia during mating is favored under specific light conditions. This has been investigated in *F. graminearum* [32,33], in which the perithecia are not formed in the dark but under light, with 4 h of daily light being enough for their optimal production. Moreover, reducing UV exposure lowers the number of perithecia. Ascospore release is also stimulated by light in this fungus [34].

### 2.2. Effect of Light on Carotenogenesis

Photocarotenogenesis is the most well-characterized light-regulated process in *Fusarium* [35]. The first studies on the effect of light on carotenogenesis were carried out on *Fusarium aquaeductuum*, which showed a gradual accumulation of carotenoids after illumination, reaching a maximum at 12 h [36]. The carotenogenetic reaction to light in this species is independent of temperature in the range of 5 to 25 °C but requires oxygenation and active protein synthesis [37]. The light-inducing effect can be partially replaced by the addition of oxidizing reagents [36,38], suggesting that the oxidation of the -SH groups plays a role in the light sensing system, which disappears when reducing agents are added [39]. However, while brief exposure to light is sufficient for photoinduction, oxidizing agents must be continuously present to maintain their stimulatory effect in addition to that of light [40], indicating different mechanisms of action. Nevertheless, the oxidizing agent *p*-hydroxymercuribenzoate had no effect on other *Fusarium* species [41].

**Figure 1:**
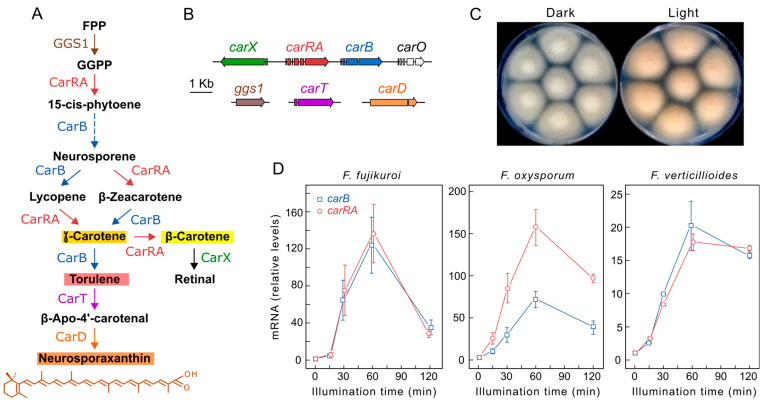
Photoregulation of carotenogenesis in *Fusarium*. (**A**) Carotenoid biosynthetic pathway. The colors of the main carotenoids that contribute to pigmentation in the mycelium are indicated in the background of their names. FPP: farnesyl diphosphate, GGPP: geranylgeranyl diphosphate. GGS1 is a prenyl transferase, CarRA is a bifunctional phytoene synthase/carotene cyclase, CarB is a desaturase, CarT and CarX are cleaving dioxygenases, CarD is an aldehyde dehydrogenase. (**B**) Genomic organization of the indicated genes in the pathway. The *carO* gene encodes a rhodopsin using retinal as chromophore. (**C**) Colonies of *F. fujikuroi* IMI58289 grown for one week in the dark or under illumination at 30 °C on minimal medium. (**D**) Light inductions of mRNA levels of the *carRA* and *carB* genes in three *Fusarium* species. Data from left to right adapted from [50] and from [42,44] (Copyright Elsevier, 2012 and 2016), respectively.

All *Fusarium* carotenoid synthesis genes have been identified [35], and the pathway is well established (Figure 1A). Three structural genes, *carRA*, *carB*, and *carX*, required for torulene, β-carotene, and retinal production, are organized in a gene cluster coregulated with a rhodopsin gene, *carO* (Figure 1B). The photoinduction of carotenogenesis in *Fusarium* mycelium grown in the dark involves a rapid increase in the transcript levels of most structural genes during the first hour of illumination, followed by an accumulation of carotenoids in the following hours, providing an orange pigmentation to the mycelium (Figure 1C). Northern blot and RT-PCR analyses of the four clustered *car* genes of *F. fujikuroi* showed a similar induction kinetics, also found for the *carT* gene, which is required for neurosporaxanthin synthesis. Similar results were obtained in *F. oxysporum* [42] and *F. verticillioides* [43,44] (Figure 1D). Recent RNA-seq data have also revealed the significant photoinduction of *ggs1* coding for a prenyl transferase [6], which has been previously underestimated [45]. The *carD* gene exhibited a lower photoresponse in *F. fujikuroi* [46], as corroborated by RNA-seq data [35]. Therefore, in *F. fujikuroi*, all carotenoid metabolism genes are regulated by light. The regulatory proteins responsible for this photoresponse are mentioned in later sections, but other proteins involved in the light signal transduction pathway may also be responsible. *Fusarium* has the predicted components of a Velvet complex, FfLae1, FfVel, and FfVel2 [47], which is connected to light regulation in other fungi [48]. The *carRA* gene is upregulated in *Fflae1* mutants, indicating a repressor function for *lae1* gene [49].

### 2.3. Effect of Light on the Production of Other Secondary Metabolites

Light modulates the production of other metabolites in addition to carotenoids. Gibberellin biosynthesis is stimulated by light in some strains of *F. fujikuroi* [24,51], although its effect is minor compared to that caused by nitrogen shortage. The influence of light on the synthesis of enniatins, cyclohexadepsipeptide antibiotics produced by different *Fusarium* species, has also been investigated. Enniatin production is enhanced by light in *Fusarium sambucinum* [52]. In other fungi, such as those of the genera *Aspergillus* or *Neurospora*, light influences the production of secondary metabolites through the Velvet VelB/VeA/LaeA complex [47,53]. In *Aspergillus*, this occurs through light controlling the VeA passage into the nucleus in response to a signal from photoreceptor proteins [54]. In *Neurospora crassa*, light promotes the degradation of the Velvet Ve-1 protein [55]. Disruption of the Velvet complex genes in *F. fujikuroi* almost completely halts the biosynthesis of gibberellins, fumonisin, fusarins, and fusaric acid [48,49], as well as conidiation. *F. graminearum* deletion mutants of the *FgVeA* and *FgVe1* genes show reduced aerial hyphal formation, as well as reduced biosynthesis of deoxynivalenol, aurofusarin, and trichotecene [56,57]. No attention has been paid, for either of the two *Fusarium* species, to the effect of light on these phenotypic changes. However, deleting the Velvet complex genes *veA*, *velB*, and *laeA* drastically reduces beauvericin production in *F. oxysporum* under light and dark conditions, in addition to affecting conidia production and morphology [58]. These mutants exhibited fewer differences in pigmentation and morphology between light and dark growth colonies than those exhibited by the wild type, confirming their connection to light regulation.

## 3. Fungal Photoreceptors

The proteins responsible for light detection and signal transmission are known as photoreceptors. They bind to small molecules called chromophores, which can absorb light and cause a conformational or chemical change in the cognate protein. This triggers a direct response or initiates a signal transduction pathway [59]. Depending on the nature of their chromophore, photoreceptors can detect light or radiation within a specific wavelength range. Thus, UV-, blue/UV-, green-, or red-light photoreceptors can be distinguished. Flavin, retinal, and tetrapyrrole chromophores are the typical fungal photoreceptor chromophores [3]. Most light responses studied in fungi are caused by the detection of blue light, although responses at other wavelengths are also known [3,60,61,62]. The main families of photoreceptors in fungi and their presence in *Fusarium* are described below. *Fusarium* genomes contain genes for ten photoreceptors (Table 1). Most of them have been studied by targeted deletion in several *Fusarium* species, while others await investigation.

## 4. Flavoproteins

Flavoprotein photoreceptors are a heterogenous group of proteins that detect blue light through the absorption of a flavin chromophore [73], bound to a LOV domain (from Light, Oxygen and Voltage). Best known classes in fungi are the WC-1 proteins of the White Collar system and VIVID proteins, but there are other LOV-containing proteins that have received poor attention in fungal photobiology.

### 4.1. White Collar Complex

The White Collar complex (WCC), the first photoreceptor system investigated in fungi, was discovered in *N. crassa* as a photoreceptor needed to induce carotenogenesis by light [74]. The WCC is composed of two proteins, WC-1 and WC-2 [75,76], which centralize all known photoresponses in this fungus, including the light control of carotenogenesis, conidiation, circadian rhythm, and the orientation of their perithecial beaks [75,77]. The photosensitivity of WC-1 relies on its LOV domain (light, oxygen, and voltage), which is a variant of the PAS domain of interaction between proteins. LOV-containing photoreceptors regulate most responses to light in fungi [3,62] using flavin-type chromophores [78]. 

A flavin is non-covalently attached to the LOV domain through hydrogen bonds, Van der Waals forces, and electrostatic interactions. Light excitation of the bound flavin, either flavin mononucleotide (FMN) or flavin dinucleotide (FAD), produces a transient covalent bond with a conserved cysteine of the LOV domain [79,80]. The formation of this link modifies the structure of WC-1 by initiating a cascade of signals in response to light. When light excitation stops, this link is slowly broken, restoring the protein to its initial fully oxidized state. WC-1 also contains two additional PAS domains [81,82] and a zinc-finger DNA-binding domain. The partner of WC-1, WC-2, is a smaller protein that lacks a LOV domain, meaning it cannot act as a photoreceptor on its own, but does have PAS and a zinc-finger domains.

**Figure 2:**
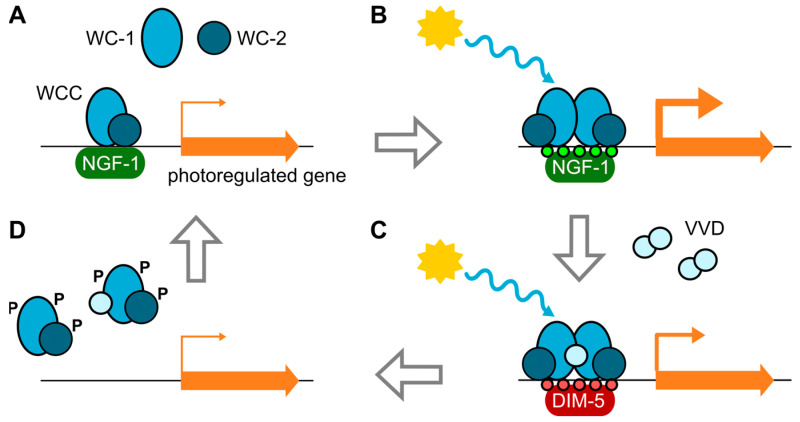
Simplified scheme of the mode of action of the White Collar complex (WCC) in *N. crassa*. (**A**) The WCC, consisting of WC-1 and WC-2, binds to the promoters of light-regulated genes in the dark. (**B**) Upon receipt of blue light, the interaction between WC-1 proteins forms WCC dimers that activate transcription through chromatin modifications (H3K14ac, small green circles) by histone acetyltransferase NGF-1. The transcription of many genes, including *vvd*, *frq*, and *sub*-1, is stimulated. (**C**) VVD protein accumulates in dimers in the nucleus and interacts with WC-1 in the WCC. New histone modification (H3K9me3, small red circles) by DIM-5 further contributes to stopping WCC activation. (**D**) WCCs dissociate from promoters and are transiently phosphorylated through PKC activity (not shown). WC-1 is partially degraded. After dephosphorylation or synthesis of new WC proteins in the dark, the cycle restarts. Adapted with permission from [1]; copyright 2016 American Society for Microbiology.

In *N. crassa*, the WCC in the dark binds to specific DNA elements mostly located in the promoters of light-regulated genes [83,84]. Light promotes the interaction of two WC1 proteins through their LOV domains, to form a WCC dimer [85]. The mechanism by which the WCC controls the transcription of target genes in response to light in this fungus involves the participation of other regulatory proteins (Figure 2) [3,62]. WCC binding mediates chromatin remodeling, facilitating RNA polymerase accessibility and transcription initiation. WC-1 mediates H3 histone acetylation (H3K14ac) by histone acetyltransferase NGF-1 [86,87], and nucleosomes are displaced after the binding of the transcription factor SUB-1 [84]. Activation of the WCC by light [88] leads to the transcriptional induction of early photoinducible genes, including VVD (VIVID, see next section). VVD is a small flavoprotein that is activated by light, binds to WCC to attenuate its activity, and leads to the phosphorylation of WC-1 and WC-2 by protein kinase C (PKC) [89]. WC-1 phosphorylation promotes its partial degradation while reducing its DNA-binding capacity [90,91]. The activity of the WCC is also determined by its interaction with the FRQ (FREQUENCY) protein [92,93], the core of the central oscillator of the circadian clock in this fungus [94,95].

Analyses in other fungi have shown orthologs of WC-1 and WC-2 in numerous species, suggesting an early evolutionary start to the regulation of responses to light [96]. They have been described in zygomycetes such as *Mucor circinelloides* or *Phycomyces blakesleeanus* [97,98]; in basidiomycetes such as *Cryptococcus neoformans* [99], *Ustilago maydis* [100], or *Pleurotus ostreatus* [101]; in ascomycetes such as *Trichoderma reesei* [102], *Botrytis cinerea* [103], or *Aspergillus nidulans* [60]. In general, fungal genomes possess a single *wc1*-like gene, although the latter has been lost in some species [96]. In contrast, some zygomycetes have several *wc-1- and wc-2*-like genes [104]. Their functions have been studied in detail in *M. circinelloides*, in which they have different specialized functions, possibly because of the loss of other photoreceptors [62]. Thus, Mcwc-1a regulates phototropism, Mcwc-1b participates in the light-independent regulatory mechanism of carotene synthesis through the ubiquitylation of the negative regulator CrgA [105], and Mcwc-1c regulates the light-dependent expression of carotenogenic genes [98]. In recent years, phenotypes related to processes not regulated by light have also been described in *wc* mutants of *N. crassa* [84]. Light-responsive transcription factors (LTFs) and their regulation by WCC have been studied in *B. cinerea*. The genes encoding BcLTF1, BcLTF3, and BcReg1 are induced by BcWcl1 [106]. BclTF1 represses BcLTF2, and the latter is repressed by the WCC. BcLTF3 represses *Bcltf2* which is also a regulator of conidiation. The deletion of *bcwcl1* causes conidiation in this fungus in both light and darkness (“always conidiation” phenotype), which correlates with increased *Bcltf2* mRNA levels [103]. The BcLTF1 ortholog in *A. nidulans*, Nsd1, plays a role in regulating the development of conidia and sclerotia [107].

#### *Fusarium* White Collar System

**Figure 3:**
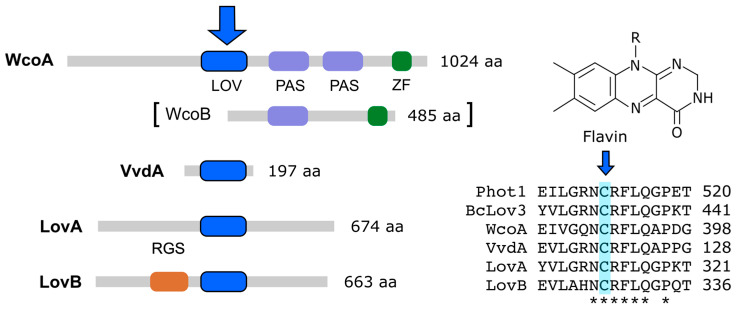
Flavin photoreceptors in *Fusarium* proteomes. Protein sizes and the major protein domains in *F. fujikuroi* are shown. The big blue arrow indicates the domain in which the chromophore, depicted on the right, is bound. WcoB is involved, as a partner of WcoA, in the WC complex. The LOV domains in LovA and LovB were identified through the NCBI domain search tool. Right: alignment of a 15-aa segment in the LOV domain of Phot1 from *Arabidopsis thaliana* (At3g45780), BcLov3 from *B. cinerea* (Bcin10g03870.1), and *F. fujikuroi* photoreceptors WcoA, VvdA, LovA, and LovB (Table 1). The conserved flavin-binding cysteine residue is shaded in blue and pointed with a small arrow. Numbers indicate the position of the last residue of the displayed segment in the corresponding protein.

*Fusarium* genomes contain an ortholog for *N. crassa wc-1, wcoA* (Figure 3). However, a more detailed study shows regulatory differences: *wc-1* expression is photoinducible in *N. crassa* while *wcoA* is hardly affected by light in *F. fujikuroi* [63]. Furthermore, their mutant phenotypes differ greatly between both genera. Secondary metabolism is affected in *wcoA* mutants of *F. fujikuroi*, which show changes in the production of gibberellins, bikaverin, and fusarins, while trichotecene synthesis and aurofusarin synthesis are affected in *F. graminearum* [64]. In addition, the *wcoA* mutants of *F. fujikuroi* have been shown to produce fewer conidia on agar or in liquid cultures, and their aerial mycelia were shown to be less hydrophobic than those of the wild-type strain [63], a phenotype also observed in the *wc1* mutant of *F. oxysporum* [65]. Photoreactivation after UV treatment was shown to be impaired, as there was no transcriptional activation of *phr1* under illumination in the *wc1* mutant in *F. oxysporum* [65] and *F. graminearum* [32].

Orthologs of the *wc-1 and wc-2* genes have also been deleted in *F. graminearum* and *Fusarium asiaticum* [32,64,66], in which phenotypes of impaired sexual reproduction, photoreactivation, and secondary metabolism have been described. No effect on virulence was reported in *F. graminearum*, but a decreased virulence in wheat, as well as defects in perithecia and ascospore formation, was shown by the *Fawc1* mutant of *F. asiaticum* [66]. Interestingly, the effect on virulence appeared to be *Fawc1*-dependent, indicating the Wc2-independent roles of this Wc1 protein. Pathogenesis was also affected in *F. oxysporum*, as indicated by the decreased virulence of the *wc1* mutant in immunosuppressed mice.

The involvement of the Velvet complex in conidiation and in the regulation of secondary metabolism and its interdependence with the WCC, as documented in other fungi [108], provides a possible explanation for the *wcoA* mutant phenotypes in *F. fujikuroi*. Interestingly, many of these alterations were independent of light. Differential phenotypes have been described for *Fawc1* mutants of *F. asiaticum* depending on whether this protein contains the LOV or the Zn-finger domains. This could explain the differential role of WC-1 orthologs, which function as a light-dependent transcription factor for some processes while functioning as a standard one in others.

Mutation of *wcoA* affects carotenogenesis to varying degrees in different *Fusarium* species, some of them in an unexpected way compared to the *N. crassa wc-1* mutant. In this fungus, whose action spectrum for light-induced carotenogenesis [109] is similar to that of *Fusarium* [110], photoinduction in mycelium is completely dependent on the WCC [3]. However, the *wcoA* mutants in *F. fujikuroi* [63] and its ortholog, *wc1*, in *F. oxysporum* [65] maintain a significantly increased carotenoid content under continuous light. However, the deletion of *fawc1 and fawc2* in *F. asiaticum* was found to prevent the induction of carotenoid biosynthesis under constant illumination [66]. The *fgwc-1* and *fgwc-2* targeted mutations in *F. graminearum* were found to cause paler pigmentation in their colonies under light, but their carotenoid content was not determined [64]. Interestingly, both mutants also exhibited higher fertility, even in unfavorable mediums for sexual development. The similar phenotypic alterations displayed by the *wc-1*- and *wc-2*-type gene mutants in the two latter *Fusarium* species support their function as a White Collar complex.

A kinetics analysis of carotenoid accumulation revealed that there are two stages in the response of *F. fujikuroi*; a rapid first WcoA-dependent response and a slower second phase of carotenoid accumulation, which was dependent on the DASH CryD cryptochrome [50] (see Section 6.1). The participation of CryD as a second photoreceptor could explain the maintenance of carotenoid accumulation in *wcoA* mutants of this species under continuous illumination.

### 4.2. VVD-Like Flavoproteins

The increase in the mRNA levels of photoinduced genes is usually transient, and after prolonged illumination is reduced or even returns to the basal levels found in the dark. This phenomenon, known as photoadaptation [111], was investigated in detail in *N. crassa*, in which it is mainly due to the counteracting effect of the VVD (VIVID) photoreceptor, a small flavoprotein with a LOV domain and an N-terminal extension of 70 amino acids involved in its dimerization [112]. Upon illumination, the WCC stimulates the transcription of the *vvd* gene and VVD protein accumulates [113]. A cysteine in VVD forms a covalent bond with a flavin after illumination, inducing a conformational change in its N-terminal domain that appears to be essential for its function [114].

If illumination persists, as is often the case of daylight in nature, the newly formed VVD can dimerize unstably and repress WCC by forming WCC–VVD complexes [115] (Figure 2) and regulating their phosphorylation [116]. The interaction of VVD with the LOV domain of WC-1 disrupts the WCC dimers and reduces their transcriptional response, leading to photoadaptation while protecting the complex from degradation [85]. The *vvd* gene itself is tightly regulated by a repressor complex consisting of the RCO-1 and RCM-1 proteins [117,118]. VVD-mediated photoadaptation also involves chromatin modifications by DIM-5, resulting in the trimethylation of histone H3 at lysine 9 (H3K9me3) (Figure 2). This was tested on the *frq* promoter, in which the *dim-5* mutation supports the role of H3K9me3 in WCC eviction as more amounts of WC-2 bind to its promoter and a greater amount of *frq* mRNA accumulates [119]. Through all of this, *N. crassa* is able to dampen the transcriptional response to light and prevent the accumulation of light-regulated mRNAs under prolonged illumination. This is evidenced by the increased accumulation of light-upregulated mRNAs in *vvd* mutants, including those of the enzymes required for carotenoid biosynthesis, leading to the more intense orange color of the mutant mycelium, hence its name [62,113].

There are VVD-like proteins in other fungi. Envoy (Env1), the *vvd* ortholog in *T. reesei*, has also been extensively studied. Env1 has a more relevant role in this fungus since its loss causes poor growth in light [120] and affects cellulase production [121]. Like *N. crassa vvd* mutants, *env1* mutants are affected in terms of their certain light-dependent responses, as well as in terms of their abilities to maintain gene photoinduction [102]. In *Trichoderma guizhouense*, the *env1* mutant exhibits much higher mRNA levels of hydrophobin genes in the light, which is explained by a lack of photoadaptation [122]. In these fungi, Env1 has emerged as a central checkpoint for the integration of nutrient sensing, light response, and development [123].

#### *Fusarium* Vivid Protein VvdA

The ortholog of *N. crassa vvd* in *F. fujikuroi* was designated as *vvdA* [67]. Its expression is strongly induced by illumination through the WcoA protein. In contrast to its *N. crassa* counterpart, *vvdA*-targeted mutation causes paler pigmentation under constant illumination due to the accumulation of less carotenoids than in the wild type. However, immediately after illumination, the *vvdA* mutant exhibits a faster carotenogenetic response to light, indicating that, as in *N. crassa*, it exerts negative control over WcoA activity [50]. The reversal of this effect after long-term illumination suggests that VvdA exerts a positive effect on a second photoreceptor. The *vvdA* mutants show morphological alterations only under illumination. Thus, when put under light, the mycelium of the *vvdA* mutants was of a higher hyphal density and was more embedded in the agar than that of the wild type or of the complemented strains [67]. Furthermore, conidial production was reduced to half in agar cultures, was strongly reduced in illuminated liquid cultures, and was restored in the complemented strain, indicating the importance of this gene in this developmental process. [67]. The essential role of VIVID proteins in conidiation under illumination has also been described in *Beauveria bassiana* [124].

### 4.3. Other Flavin Photoreceptors

Fungal genomes frequently contain genes that encode other LOV flavin-binding domains in addition to those described above. Two types have been distinguished, some with a regulatory domain of G protein signaling (RGS) and others without recognizable domains with possible regulatory functions [61,125]. Genes for both types of proteins exist in fungi, such as *Magnaporte*, *Botrytis*, or *Fusarium*, but are not found in other well-known fungi, such as *Aspergillus* or *Neurospora*. These putative fungal photoreceptors have received very little attention. An exception is the RGS-domain flavoprotein RGS5, which was investigated in *Magnaporthe oryzae*. RGS5 was shown to interact with two of the three Gα subunits of this fungus, and the *Morgs5* deletion mutant was shown to contain higher intracellular cAMP levels, although no apparent phenotypic alterations were shown [125]. *F. fujikuroi* contains two putative photoreceptors that we call LovA and LovB (Table 1), the second with an RGS domain (Figure 3). Although they are smaller, both proteins show similarity to plant phototropins. Thus, 145 of the 674 LovA residues and 154 of the 663 LovB residues have coincident positions in the *Arabidopsis thaliana* Phot1 996-aa polypeptide (Uniprot O48963).

## 5. Cryptochromes

The proteins of the cryptochrome–photolyase family are a heterogenous group of photoreceptors that absorb radiation from the UV-A/blue spectral range via two chromophores, FAD and 5,10-methenyltetrahydrofolate (MTHF). They are present in all the main taxa and are divided into 10 subfamilies [126], five corresponding to photolyases (PHR) and five to cryptochromes (CRY), the latter being of plant origin and the rest being generally found in animals. Photolyases act as light-driven DNA-repair enzymes [127], and cryptochromes are often involved in signal transduction. Cryptochromes consist of the typical N-terminal region of a photolyase (PHR), followed by a carboxyl terminal domain of variable length, which is involved in signal transduction. A special subgroup are the DASH-cryptochromes (*Drosophila*, *Arabidopsis*, *Synechocystis*, and *Homo*), which exhibit both functions [128]. In general, CRY-DASH proteins bind single-stranded DNA, with some exceptional cases in which they bind double-stranded DNA [129] to perform repair functions typical of photolyases [129].

Cryptochromes belonging to the plant type and DASH cryptochrome groups are generally found in fungi, and they also contain canonical photolyases that exert repair functions. Nevertheless, in some zygomycetes that lack photolyases, the repair function is carried out by DASH cryptochromes [129,130]. Compared to other taxa, there is not much information on fungal cryptochromes and their signaling mechanisms [3], although several mutants have been described. In *A. nidulans*, CryA is closer to CPD (Cyclobutane Pyrimidine Dimer) photolyases and has repair functions [131], but also represses sexual development in the presence of light. In *N. crassa*, the Cry-DASH CRY protein is involved in circadian rhythm regulation and binds to DNA and single- and double-stranded RNA but shows no evidence of photolyase activity in vivo [132,133]. *B. cinerea* has two members of the family with different functions, BcCRY1, a CPD photolyase, and BcCRY2, a Cry-DASH protein that is dispensable for photorepair but that plays a role of a conidiation repressor [134]. Fungal Cry-DASH proteins in other species, *Sclerotinia sclerotiorum* and *Cordyceps militaris*, regulate very different processes, including development or secondary metabolite production [135,136].

### Fusarium Cryptochromes CryD and CryP

**Figure 4:**
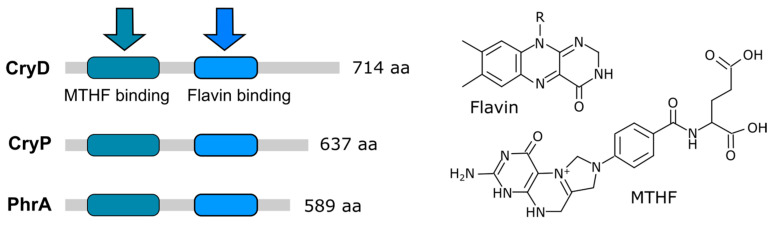
Photoreceptors of the cryptochrome/photolyase family in *Fusarium*. Protein sizes and chromophore binding domains in *F. fujikuroi* are shown. The arrows indicate the binding of the chromophores that are depicted on the right. MTHF: 5,10-methenyltetrahydrofolate.

**Figure 5:**
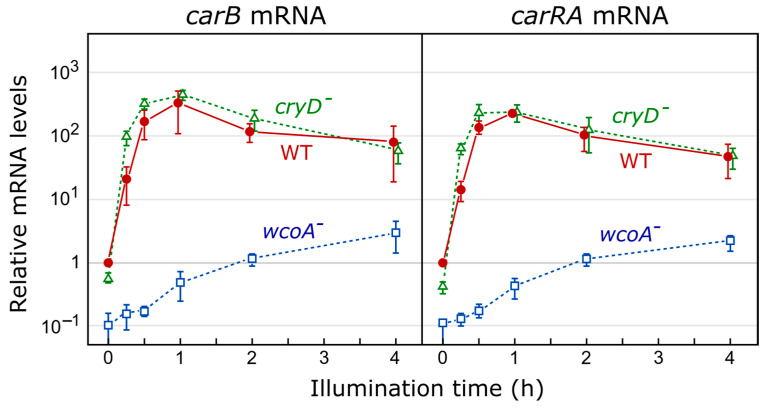
Effect of *wcoA* or *cryD* mutations on light induction of mRNA levels of the *carRA* and *carB* genes in *F. fujikuroi*. Cultures grown in shake flasks in the dark were transferred to Petri dishes, adapted to these conditions for 4 h, and then illuminated for the indicated intervals. mRNA levels are referred to those of the wild type (WT) in the dark. Adapted from [137,138].

*Fusarium* genomes have two cryptochrome genes: one for a plant-type cryptochrome and one for a DASH cryptochrome (Figure 4). The DASH cryptochrome CryD from *F. fujikuroi* has received special attention, and its photoactivity has been demonstrated biochemically [68]. The expression of *cryD* is strongly stimulated by light via WcoA, and its targeted mutation produces different phenotypic alterations only under illumination, such as the formation of macroconidia under nitrogen starvation or bikaverin production at 30 °C [51]. Furthermore, CryD seems to be responsible for the long-term photoinduction of carotenogenesis, as indicated by the slower accumulation of carotenoids after the prolonged incubation under light of *cryD* mutants [50]. However, *wcoA* mutants accumulate carotenoids very slowly from the onset of illumination, and their total levels do not reach those of the wild type. The photoinduction of the structural genes for carotenogenesis in the *wcoA* and *cryD* mutants (Figure 5) is consistent with different mechanisms of action for the encoded photoreceptors [50]. The *cryD* mutant has exhibited wild-type photoinduction of the *car* structural genes. However, in the *wcoA* mutant, the mRNA levels were much lower than those in the wild-type strain even in the dark, but still detectably increased after illumination. The induction of carotenogenesis by the alternative photoreceptor, presumably CryD, must take place via a transcription-independent mechanism that remains to be elucidated.

No information is available on the function of the plant-type *Fusarium* cryptochrome, but data from RNA-seq studies show that its expression is also induced by light [6]. On the other hand, photoreactivation due to UV damage is carried out in *Fusarium* by Phr photolyase, whose expression is also light-dependent [6,69]. Furthermore, it has been found to be highly expressed in the early stages of germination [139].

## 6. Rhodopsins

Green light can be sensed in fungi by retinal-binding opsins, a family of integral membrane proteins also known as rhodopsins [140]. Opsins have a highly conserved three-dimensional structure, consisting of seven transmembrane helices. Those capable of detecting light attach a retinal molecule as a chromophore by covalent bonding to a lysine residue located in the third transmembrane domain. These proteins were initially discovered in archaea through the study of bacteriorhodopsin, a light-driven proton pump, and were subsequently found in other taxa, including fungi [141]. Three classes of opsins are found in fungi, two of which act as photoreceptors: the first class has a slow photocycle with low proton pump activity, such as NOP-1 in *N. crassa* [142]; the second class is characterized by strong green light-dependent proton pump activity, such as CarO in *F. fujikuroi* [143]; the third class lacks the retinal binding residue and is therefore not expected to be photoactive [3]. The genomes of many fungi encode other opsin-related proteins that lack the lysine residue for chromophore binding, the functions of which remain enigmatic [144]. In *N. crassa*, NOP-1 is involved in the regulation of developmental processes, possibly through the control of the oxidative state of the cell [142], while the CarO-type rhodopsin has been found only in plant-associated fungi and has a role in germination and infection in *F. fujikuroi* [70]. *U. maydis* has three rhodopsin genes, two with proton pump activity and a third without such activity that is only expressed during plant infection [145]. The signal transduction pathway for fungal rhodopsins is still unknown. The presence of rhodopsin genes in fungal genomes is very variable, and phylogenetic studies suggest the occurrence of horizontal transfer events followed by duplications and losses, presumably leading to their current functional diversification [142].

### 6.1. Fusarium Rhodopsins CarO and OpsA

**Figure 6:**
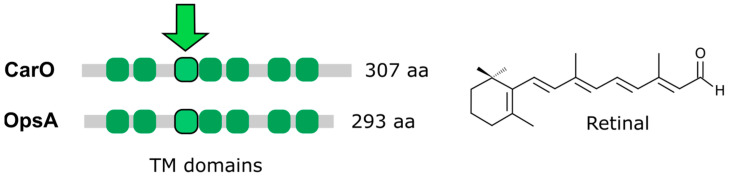
Rhodopsins in *Fusarium* proteomes. Protein sizes and transmembrane domains in *F. fujikuroi* rhodopsins are shown. The two proteins bind to the retinal chromophore. The arrow indicates the transmembranal (TM) domain in which the chromophore is covalently attached to a conserved lysine residue.

*Fusarium* has two rhodopsins, which have been studied in *F. fujikuroi* (Figure 6). The rhodopsin gene *carO* is linked to the *carRA*, *carB*, and *carX* genes in the *car* gene cluster [71] and its transcription is induced by light. The CarO protein is a proton pump that efficiently responds to green light and whose activity is enhanced in the presence of the plant hormone indole-3-acetic acid [70,143]. A targeted mutant of the *carO* gene lacks apparent phenotypic changes [71], but germination analyses of conidia have shown the faster development of light-exposed germlings in the *carO* mutant than in the control strain. Furthermore, a visualization of YFP-labeled CarO showed its presence in the conidia produced by illuminated mycelia. Orthologs of this rhodopsin have been found in many plant-associated fungi, suggesting its possible role in plant infection. In addition, rice plants infected with the *carO* mutant showed more severe *bakanae* symptoms [70], indicating the possible role of rhodopsin CarO in controlling plant infection.

The second *Fusarium* rhodopsin gene, *opsA*, is orthologous to the *N. crassa nop-1* gene. Mutation of *opsA* gene resulted in no visible phenotypic alterations, including the carotenoid content, although a moderate decrease in *car* gene mRNA levels was shown [72]. The *carO* and *opsA* genes were differentially expressed during sexual reproduction in perithecia. The *carO* gene was down-regulated in a mutant of the sexual locus *mat1-2-1* in *F. verticillioides* [146], and its transcript levels increased throughout the period of perithecia maturation in *F. graminerarum* [142]. The *N. crassa* Δ*nop-1* mutant initiates sexual development earlier than the wild type does, suggesting the role of OpsA in the sexual cycle. Moreover, comparative transcriptomics showed the increased expression of genes involved in the response to oxidative stress in the *nop-1* mutant [142], indicating that Nop-1 could play a role in the regulation of asexual–sexual development in response to different environmental cues, including light and reactive oxygen species (ROS).

*Fusarium* has a third gene in the opsin family, *hspO*, which has not been investigated. The HspO protein is predicted to lack the conserved retinal binding lysine and is therefore assumed to be a non-photoactive opsin [72]. Some of these opsin-related proteins have been associated with stress responses and could function as chaperones [141], but their mechanisms of action remain unknown.

## 7. Phytochromes

Red and far-red light is detected in fungi by phytochromes, which use a linear tetrapyrrole (bilin) chromophore that is autocatalytically linked to the apoprotein through a conserved cysteine residue [147]. Originally described in plants, this type of photoreceptor was first discovered in fungi in *A. nidulans* [148] and *N. crassa* [149]. Phytochrome genes are common in ascomycetes [150] and basidiomycetes [62] but have not been described in zygomycetes. Some fungi contain more than one phytochrome, indicating functional diversification. This is the case for *Monilinia laxa*, which has three phytochromes with different transcriptional regulations [151]. Fungal phytochromes (Fph) contain a photosensor module at the amino terminus consisting of a PAS domain, which is also present in bacterial phytochromes. They also contain a GAF domain (cGMP phosphodiesterase/Adenylate cyclase/FhIA) that exhibits bilin lyase activity, which includes the conserved cysteine residue that binds to the chromophore, and a PHY domain unique to this class of photoreceptors. In addition, they contain a domain related to histidine kinases or HKRD and, depending on the species, a regulatory module in their highly variable carboxyl termini [147]. The bilin-related chromophore of the FphA phytochromes of *A. nidulans* and *Alternaria alternata* is biliverdin or a very similar derivative [148,152]. The synthesis of this chromophore and its coupling to FphA has been investigated in *A. alternata*, in which FphA interacts with two mitochondria-attached heme oxygenases involved in chromophore synthesis [152]. This interaction mediates the transfer of the chromophore to the phytochrome.

In *A. nidulans*, the phytochrome plays a central role, regulating the balance between asexual and sexual development and the germination process, as well as secondary metabolite biosynthesis [148,153]. The mechanism of action has been investigated in detail in this species, in which it induces the HOG signaling pathway in the cytoplasm, modulating the activity of the phosphotransferase protein YpdA [154], and interacts in the nucleus with transcription factors and enzymes that are necessary for chromatin modification [60,155,156]. In the plant pathogen *A. alternata*, red and blue light have inhibiting and stimulating effects on sporulation, respectively, through the participation of the FphA phytochrome and the LreA WC protein in coordination with the high-osmolarity glycerol (HOG) mitogen-activated protein (MAP) kinase pathway [157]. In addition, both photoreceptors modulate other processes, such as spore germination and ROS alleviation, by catalase and superoxide dismutase activity. The HOG signaling pathway also participates in the initial stage of fruit colonization, indicating the role of phytochrome in pathogenesis [158]. Phytochromes are also involved in the modulation of the activity of the WCC and in the transcription of some of their genes in several fungi, including *N. crassa* [3,60,62,134]. Phytochromes have also been described as temperature sensors in plants, bacteria, and fungi [159,160,161], which makes evolutionary sense considering that, in nature, exposure to sunlight is usually associated with an increase in temperature.

The important role of red or far-red light detection via phytochromes in *A. nidulans* [162] or *A. alternata* [157] contrasts with the prevalence of blue light detection in other fungi. There are ecological niches that allow a greater abundance of certain types of light, such as far-red light or green light in plant environments [163,164], which may explain the greater role of phytochromes in some species and their participation in key developmental processes, such as sporulation. However, the detection of specific light wavelengths may be irrelevant in other environments, in which blue light photoreceptors may function more efficiently.

Fusarium *Phytochrome FphA*

**Figure 7:**
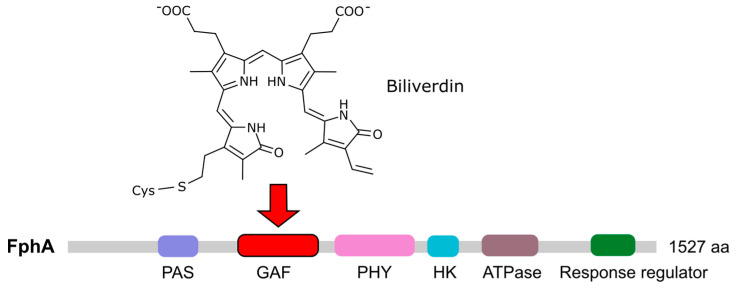
Chromophore and domain structure of the *Fusarium* phytochrome. The *F. fujikuroi* coding gene *fphA* (*FFUJ_05887*) was identified due to its sequence similarity with *F. graminearum Fgfph* (*FGSG_08608*), which has been previously described [32]. The arrow indicates the domain in which the chromophore is expected to bind to a conserved cysteine residue.

The role of the only phytochrome (Figure 7) found in *Fusarium* genomes [24] has been analyzed in *F. graminearum* [32]. The deletion of *fgfph* produced no visible phenotypic alterations, including growth, pigmentation, virulence, or fertility. However, in vivo protein–protein interaction assayed by split luciferase complementation showed that FgFph interacts with FgWc-1 as well as with the Velvet complex proteins FgVeA and FgLaeA. As already stated, the Velvet complex coordinates light perception and development and secondary metabolism in many fungi [47]. The interaction of FgFph with WC-1 and the Velvet complex suggests its indirect role in Velvet-mediated processes, possibly as an accessory photoreceptor. Furthermore, illuminating *F. fujikuroi* mycelium with red light appreciably induces the expression of at least some blue-light regulated genes, including *carRA* [50]. Although the photoinduction of carotenoids depends mainly on WcoA, this observation indicates the participation of a phytochrome in the photosensory system that regulates this response.

## 8. Study of Photoreceptor Functions through Global Transcriptomic Tools

The effect of light on fungal transcriptomes has been investigated in several organisms with different techniques [62,165], and these investigations usually conclude that light affects the mRNA levels of hundreds of genes. In *A. fumigatus*, a microarray assay was used to investigate the effect of 15, 30, 60, and 120 min of light exposure, and, based on a 1.5-fold change that was observed, it was concluded that 2.6% of the genes were regulated by light in this species [166]. In *P. blakesleeanus*, large-scale cDNA sequencing led to an estimate that, in about 5.2% of the genes, mRNA levels changed at least five-fold after 30 min of illumination [167]. A study conducted in *Trichoderma atroviride* found 246 and 215 genes upregulated and downregulated after a 30 min light pulse [168], which make up about 3.9% of its genome. These works laid the foundation for functional studies of photoreceptors, verifying the effect of mutations in the responsible genes.

Analyses of photoreceptor functions through global transcriptomic studies have mainly focused on the WC system. In *N. crassa*, about 5–6% of genes were differentially expressed in the wild type during the first 90 min after light onset, a response largely mediated by the WC complex [4]. A comparison of microarray expression data after eight illumination intervals, from 5 to 240 min, revealed at least two classes of induction patterns, leading to the classification of early responsive and late-responsive genes. Essentially, all photoresponses were lost in the *wc-1* and *wc-2* mutants. A subsequent and more precise RNA-seq study of the same fungus showed 31% of genes with at least a two-fold change in mRNA levels [83]. Different induction patterns were observed and separated into four clusters, although the classifications of early and late responsive genes were conserved. This drew attention to the occurrence of a large set of light-repressed genes, none of which showed early repression. WC-1 can also act as a repressing protein through a light responsive GATA element that has been found to be involved in binding to form a repressing complex in the dark [169]. Nevertheless, the lack of fast downregulating responses suggests that the repressive effects could have been caused by other negative regulators, which would have been activated earlier by the WC-1.

Experiments combining the effect of light on the transcriptome with the effect of mutations in the *wc* genes have also been carried out in other fungi. Microarray data from *T. reseei* showed that 2.8% of genes were differentially expressed under continuous illumination compared to a dark control, with a trend towards upregulation [7]. Surprisingly, the lack of the WC complex altered the adaptation to constant illumination, as the number of genes differentially expressed in light increased to about 9% in the mutants of the *wc-1* and *wc-2* orthologs *brl1* and *brl2*. A new RNA-seq study of the effect of a 30 min light pulse in *T. atroviride*, using a different bioinformatic analysis for differential expression, found 97 and 38 genes upregulated and downregulated in the wild type, of which only 3 from each class maintained photoregulation in the *blr-1* mutant, indicating that this WC protein mediates most of the photoresponses in this species [170]. This study of *T. atroviride* was extended to mutants for the ENVOY, CRY-1, and CRY-DASH photoreceptors, but their roles in photoregulation were subsidiary to Blr1, since the transcription of their genes was activated by light through this photoreceptor. In *Sordaria fimicola*, the effect of 15 min and 45 min of illumination on the transcriptome was analyzed in the wild-type strain and in a defective *sfwc-1* mutant. Unexpectedly, of the 874 light-regulated genes, only 466 lost photoinduction in the mutant, suggesting the participation of another blue-light photoreceptor [171]. In other fungi, the WC system plays less prominent roles. In *A. nidulans*, the effect of different light wavelengths on the transcriptomes of mutants of the LreA Wc-1 photoreceptor and the FphA phytochrome genes showed that red light exerts a greater influence than blue light, and that FphA is the main photoreceptor [162]. The loss of photoinduction of most of the red light regulated genes in a SakA kinase mutant confirmed the FphA activity in the Hog MAP kinase pathway, a result that confirmed previous molecular observations [154]. Interestingly, both LreA and FphA play regulatory functions in the dark as well, and many of the responses to blue light require a functional phytochrome.

Light is less influential in other fungi, such as in *U. maydis*, in which only 60 genes were found to be induced by light, all of which were dependent on the *wco1* gene [100]. Other fungi, however, are apparently insensitive to light; in the basidiomycete fungus *C. neoformans*, only one gene was significantly affected under illumination [172]. Taken together, the different results show the considerable variability of the effects of mutations in the White Collar genes of different species.

### 8.1. Effect of wcoA Mutation on the Fusarium Transcriptome

**Figure 8:**
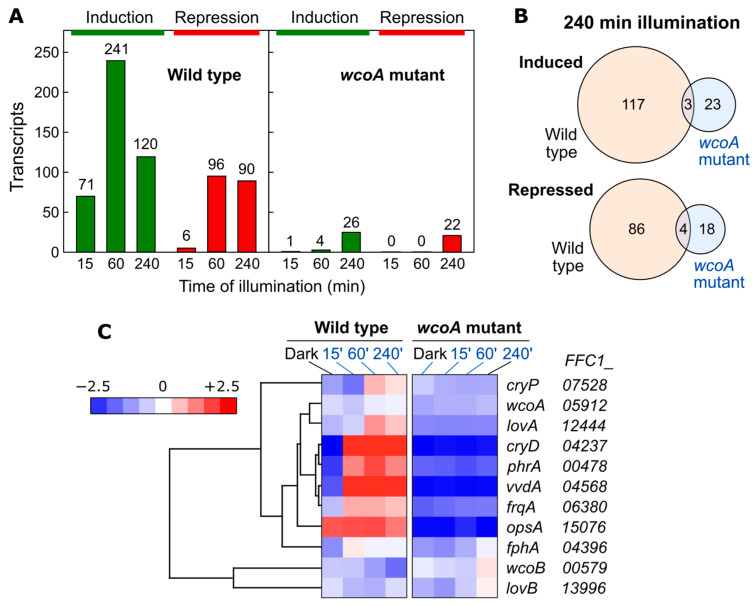
Effect of illumination on wild-type (FKMC1995) and *wcoA* mutant transcriptomes. (**A**) Numbers of genes upregulated (green) or downregulated (red) after 15, 60, or 240 min of illumination using a four-fold threshold. (**B**) Venn diagrams of genes induced (above) or repressed (below) after 240 min illumination in the wild type and *wcoA* mutant. The intersection shows the coincident genes in both strains. (**C**) Hierarchical heatmap for the effect of different illumination intervals on transcript levels for photoreceptor genes in wild type and *wcoA* mutant. The *FFC1_* numbers of the genes in the genome annotation are indicated on the right. The *N. crassa* frequency protein orthologous gene (*frqA*) is also included because of its connection to light regulation. Data adapted from [138].

As shown in Section 2.2, the most investigated light response in *Fusarium* is the photoinduction of carotenoid synthesis [35,63]. The loss of function of the *wcoA* gene produces a drastic decrease in the mRNA levels of the carotenogenesis genes, both under light and in the dark, but it does not prevent their induction by light (Figure 5). The phenotype resulting from the mutation is, however, highly pleiotropic. Diverse morphological and metabolic changes are observed, including alterations in the synthesis of different secondary metabolites, suggesting the complex regulatory role of WcoA. To further investigate the diversity of functions of this photoreceptor in this fungus, the impact of the *wcoA* mutation on the transcriptome was recently analyzed by RNA-seq. Total RNA samples of a wild-type and a *wcoA* mutant were compared either in the dark or under illumination for 15, 60, or 240 min [138]. Using changes in mRNA levels greater than 4-fold as part of the criteria for effects on regulation, 298 light-activated genes and 160 light-repressed genes were observed after at least one of the three illumination intervals (Figure 8A). Photoactivated genes were distributed in those with fast, intermediate, and slow responses, while only intermediate and slow responses were found among photorepressed genes. Most of the responses to light were lost in the *wcoA* mutant. Interestingly, 41 genes that were not regulated by light in the wild type showed slow light-dependent responses in the mutant (Figure 8B). Therefore, WcoA is the main photoreceptor for responses to light at the transcriptional level in this fungus.

WcoA is required for the photoinduction of other regulatory proteins, including several photoreceptors, confirming previously obtained results (Figure 8C). The strong photoinduction of the *genes* for cryptochrome CryD, flavoprotein VvdA, rhodopsin CarO, and photolyase PhrA is lost in the *wcoA* mutant. WcoA also participates in the photoinduction of the *F. fujikuroi* ortholog of the *frq* (*frequency)* gene of *N. crassa*, which is involved in the circadian rhythm and not yet studied in *Fusarium*. The genes for the other *F. fujikuroi* cryptochrome, which we call CryP genes, and flavoprotein LovA also show that photoinduction is lost in *wcoA* the mutant. However, *lovB* is hardly affected by light or by the *wcoA* mutation. Thus, WcoA plays a central role in *Fusarium* photobiology, not only through its direct action on many photoregulated genes, but also through its regulation of other photoreceptor genes. Of particular interest is the case of the OpsA rhodopsin gene, the mRNA levels of which are barely affected by light but drop considerably in the *wcoA* mutant both in the light and in the dark.

**Figure 9:**
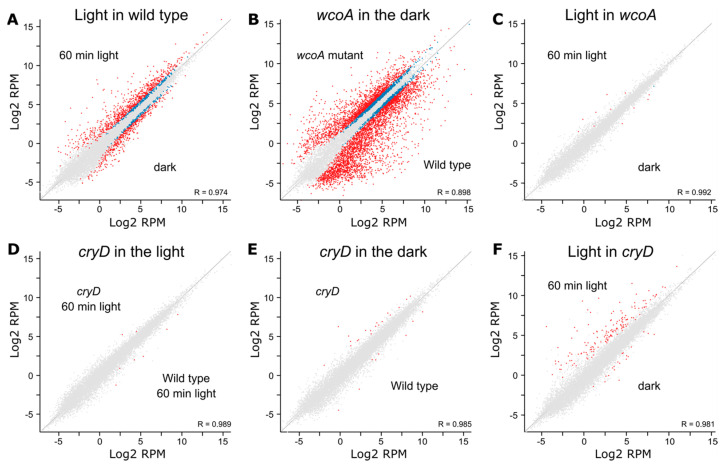
Scatter plot representations of the effect of *wcoA* or *cryD* mutations on the transcriptomic response under 60 min illumination. Differentially expressed genes according to the DESeq2 analysis of the Seqmonk program are indicated in blue. Genes that exceed log2 values of ±2 are indicated in red. (**A**) Effect of light on the wild type. (**B**) Effect of *wcoA* mutation in the dark. (**C**) Effect of light on the *wcoA* mutant. (**D**) Effect of *cryD* mutation after 60 min of illumination. (**E**) Effect of *cryD* mutation in the dark. (**F**) Effect of light on the *cryD* mutant. Data from [137,138].

The influence of the *wcoA* mutation on the *opsA* mRNA content draws attention to the most striking feature of the effect of WcoA on the transcriptome, which is that the most extensive effects of its mutation are light-independent (Figure 9). In approximately 16% of the *F. fujikuroi* genes, mRNA levels changed at least fourfold when comparing the wild type to the *wcoA* mutant in the dark or after different light exposures. The genes with decreased mRNA levels in the *wcoA* mutant (2843 genes in the sum of all conditions) predominated over those with increased mRNA levels (1297). As would be expected from the enormous number of them, the affected genes are associated with a wide variety of functions, including the production of different secondary metabolites and hydrophobins. Secondary metabolite pathway genes are normally clustered in the genome. Most of the genes of the clusters for the synthesis of fusaric acid, fusarin, gibberellin, and echisetin/trichotecin were found at much lower levels in the *wcoA* mutant than in the wild type. However, the opposite pattern was observed in the bikaverin synthesis cluster. Therefore, WcoA acts, directly or indirectly, as an activator or as a repressor of several secondary metabolism clusters.

The massive transcriptomic change found in the *wcoA* mutant in the dark, in addition to its central role as a photoreceptor responsible for most transcriptional responses to light in this fungus, points to the role of WcoA as a master regulatory protein in *F. fujikuroi*. These results were obtained with a *wcoA* mutant obtained from the wild-type strain FKMC1995. Ongoing experiments with a different wild-type strain, IMI58289, point to regulatory differences even between strains of the same species (J. Marente, personal communication).

### 8.2. Effect of cryD Deletion on the Fusarium Transcriptome

**Figure 10:**
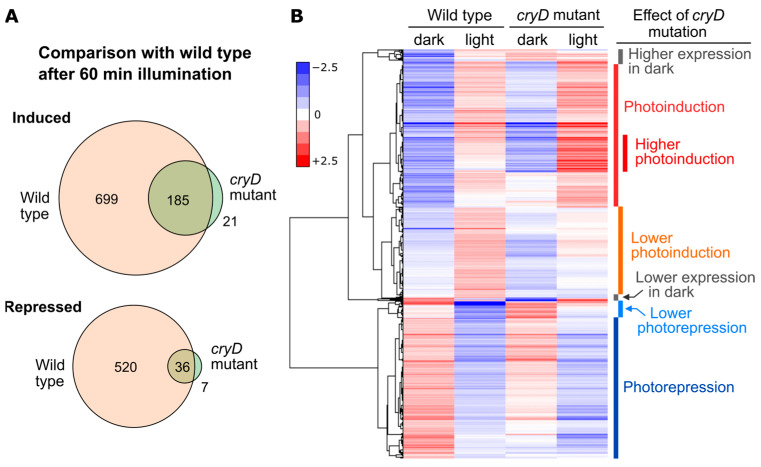
Effect of illumination on wild-type and *cryD* mutant transcriptomes. (**A**) Venn diagrams of the genes induced (above) or repressed (below) after 60 min illumination in the wild-type strain and in the *cryD* mutant (SG237) using a two-fold threshold. The intersection shows the coincident genes in both strains. (**B**) Hierarchical heatmap of genes induced or repressed after 60 min of illumination in the wild strain or in the *cryD* mutant. On the right are the groups, shown according to the effect of the *cryD* mutation. Data from [137].

In contrast to the effect of *wcoA* mutation, the *cryD* mutant exhibited patterns of photoinduction of the genes *carRA* and *carB* that were similar to those of the wild type (Figure 5). The influence of the CryD photoreceptor on the transcriptome was analyzed by studying a *cryD* mutant in comparison to the wild-type strain FKMC1995 in the dark or after 1h of illumination [137]. Experiments were performed in parallel to those on the *wcoA* mutant, summarized in Section 8.1. The number of photoregulated transcripts decreased in the *cryD* mutant (Figure 9A,D). Only 206 genes were photoinduced at least 2-fold in contrast to 884 genes in the wild-type strain under the same conditions (Figure 10A). The reduction in photorepression was more noticeable, with lower mRNA levels being found for 56 genes after illumination in the mutant versus 556 genes in the wild-type strain. By analyzing the 1549 photoregulated transcripts in either of the two strains on a heatmap, we concluded that most of the genes that showed photoinduction or photorepression in the wild-type strain also showed photoinduction or photorepression in the *cryD* mutant, but that many of them exhibited changes in response intensity (Figure 10B). This is not unexpected, considering that the major photoreceptor responsible for light regulation is WcoA. However, the data suggest the role of CryD as an accessory photoreceptor that modulates the degree of photoresponsivity of many genes.

Using the two-fold change (log2 = 1) observed as a threshold, 230 genes were differentially expressed in the dark in the *cryD* mutant compared to the wild-type strain, 154 of which were upregulated and 76 of which were downregulated. Similar numbers of affected genes were observed after one hour of illumination with 216 differentially expressed genes (DETs), of which 135 were upregulated and 81 were downregulated. When the effect of the *cryD* mutation was compared to that of the *wcoA* mutation under the same threshold, the number of WcoA-dependent DETs was much higher, this number being 3333, compared to the number of CryD-dependent DETs, which was 382. Approximately half of them (138 in the dark and 114 in the light) were shared between the mutants for the two photoreceptors. Nevertheless, 92 and 102 of the transcripts were specific for the *cryD* mutation.

Previous phenotypic analyses of the *cryD* mutant suggested the participation of this photoreceptor in the control of secondary metabolism, since they indicated alterations in the coloration pattern under light, which were mainly due to the accumulation of bikaverin [51]. However, although significant changes in mRNA levels were observed for some genes involved in secondary metabolism, those of the bikaverin cluster did not reach the two-fold change in transcript levels in the dark or after 60 min of exposure to light. The discovery of RNA molecules bound to this protein [68], unlike the other cryptochromes described, was proposed as the basis for the possible mechanism of non-transcriptional regulation.

Although CryD is presumably not a DNA binding protein, the number of affected genes in the mRNA content of the *cryD* mutant is notable. This could be due to the indirect effects on other regulatory proteins or due to changes in the stability or half-life of the mRNA. However, the drop in the number of photoinduced genes in the *cryD* mutant consolidates its role as a light-dependent regulatory protein, which is consistent with its function as a photoreceptor. Moreover, many genes that maintain their photoregulation exhibit a less prominent response [137]. Therefore, the data suggest that CryD modulates the intensity of the photoresponse of many genes. The opposite effect was also observed: an analysis of the clustering of mean values revealed a group of genes with higher photoinduction in the *cryD* mutant than in the wild-type strain. This group included several genes related to photoreception and the gene *frq*, indicating a similar possible influence of CryD on the circadian rhythm to that in *N. crassa* [132].

## 9. Concluding Remarks

The abundance of photoreceptor genes in fungi reveals the importance of light as a modulating signal for different activities. Species of the *Fusarium* genus are no exception, and the evolutionary conservation of photoreceptor genes indicates their relevant roles in the life cycle of these species and in their ability to compete and survive in environment.

**Figure 11:**
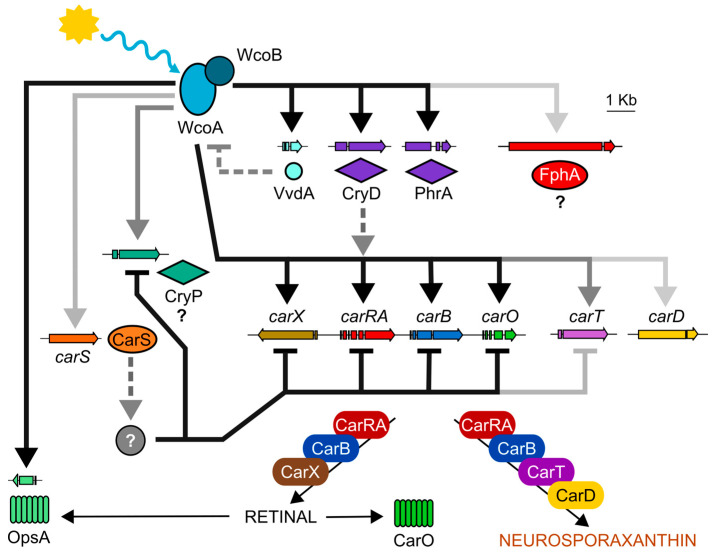
Summary of the regulatory relationships between photoreceptors and carotenogenic genes in *F. fujikuroi* according to transcriptomic and experimental data from previous works. The intensity of the arrows represents the impact of each element on their targets. CarS is a RING-finger protein that downregulates the carotenoid pathway. Dashed lines indicate post-transcriptional effects. The grey element corresponds to a hypothetical regulator that is not yet known.

As already observed in *N. crassa*, a well-known model in fungal photobiology, the White Collar system plays a central role in light regulation in *Fusarium* and also modulates other photoresponses. Noteworthily, it controls light induction of gene transcription for four of the other photoreceptors, the CarO rhodopsin, the VvdA flavoprotein, the CryD cryptochrome, and the PhrA photolyase (Figure 11). Sun exposure is accompanied by collateral damage, including exposure to UV radiation, which makes biological sense in terms of the light induction of the photolyase gene, which is responsible for repairing DNA damage caused by UV rays. With respect to the rest of the photoreceptors, progress has been made in assigning their functions, but much remains to be discovered. In the case of OpsA, CryP, or FphA, no data are available yet, although according to information on other fungi, the latter may be part of a Velvet complex, for which *Fusarium* has all the components.

The list of photoreceptors described in Table 1 is not necessarily exhaustive. Another type of blue-light photoreceptors, BLUF proteins (from “blue-light using FAD”), have been found in *Ustilago* and related species, but their functions have not yet been studied [100], and we did not find orthologs in the available *Fusarium* genomes.

Carotenoids are easy to trace externally, which explains the abundance of studies on the photoinduction of carotenogenesis in *Fusarium*. In addition, these pigments are of biotechnological interest, since they protect against oxidative damage, with neurosporaxanthin being especially effective in this regard [173]. A side effect of light is the generation of photooxidative damage, which gives biological significance to the light regulation of the synthesis of these pigments. The available information reveals a regulatory network that is depicted in Figure 11. Like in *N. crassa*, the main regulator of the light induction of carotenogenesis is the White Collar complex, with the counter involvement of VvdA. However, the available data indicate the participation of at least the CryD cryptochrome, and the participation of other photoreceptors currently under investigation, such as the CryP cryptochrome, is not ruled out. For reasons yet to be elucidated, *Fusarium* shows unexpected complexity in the photoregulation of this pathway, which most likely extends to other less externally visible light-regulated processes, such as the controlling of the sexual cycle or sporulation.

## Figures and Tables

**Table 1:** Genes for photoreceptor proteins in the genomes of three *Fusarium* species.

Group	Chromophore ^1^	Protein ^2^		References ^3^	*F. fujikuroi* ^4^	*F. oxysporum* ^5^	*F. graminearum* ^6^
Flavoproteins	Flavin (blue)	WCC ^7^	WcoA	[32,63,64,65,66]	*FFUJ_13691*	*FOXG_03727*	*FGSG_07941*
			WcoB	[32,65,66]	*FFUJ_00530*	*FOXG_01037*	*FGSG_00710*
		VvdA		[50,67]	*FFUJ_06055*	*FOXG_03254*	*FGSG_08456*
		LovA		-	*FFUJ_11713*	*FOXG_12253*	*FGSG_02972*
		LovB		-	*FFUJ_08848*	*FOXG_09176*	*FGSG_04991*
Cryptochrome photolyase family	MTHF/flavin (blue)	CryD		[50,51,68]	*FFUJ_05732*	*FOXG_03570*	*FGSG_08852*
		CryP		-	*FFUJ_03105*	*FOXG_02060*	*FGSG_06765*
		PhrA		[69]	*FFUJ_00436*	*FOXG_01134*	*FGSG_00797*
Rhodopsins	Retinal (green)	CarO		[70,71]	*FFUJ_11804*	*FOXG_12142*	*FGSG_03064*
		OpsA		[70,72]	*FFUJ_02352*	*FOXG_15406*	*FGSG_07554*
Phytochromes	Biliverdin (red)	FphA		[32]	*FFUJ_05887*	*FOXG_03424*	*FGSG_08608*

^1^ Color of absorbed light is indicated in parentheses. ^2^ Protein denomination in *F. fujikuroi*. ^3^ References to studies in which the gene or protein was investigated in a *Fusarium* species. ^4,5,6^ Gene denominations in genome annotations. ^4^ Strain IMI58289. ^5^ Strain 4287. Formae specialis *lycopersici*. ^6^ Strain PH-1. ^7^ WCC: White Collar complex. WcoA forms a complex with WcoB, in which WcoA is the light-detecting component.

## Data Availability

RNA-seq data in Figure 8, Figure 9 and Figure 10 are available in NCBI’s Gene Expression Omnibus repository under GEO Series accession numbers GSE159533 and GSE223375.
